# Expanding clinical and variant spectrum of *CBX1* -related syndrome: Report of three novel cases

**DOI:** 10.64898/2026.09.28.26363820

**Published:** 2026-09-28

**Authors:** Graciela Carrillo, Shuhei Fujino, Harrison Higgs, Anita Kabahizi, Randy Le, Rachel Rabin, John Pappas, Amy Crunk, Kara Ranguin, Jérémie Mortreux, AURAGEN consortium, Marilyn Lackmy, Carlos A Gadia, Matthew Hoi Kin Chau, Jill A. Rosenfeld, Xi Luo, Kosuke Izumi

## Abstract

*CBX1* -related syndrome is characterized by developmental delay, hypotonia, autistic features, and mild dysmorphic features. This syndrome is caused by heterozygous missense variants in *CBX1* , which encodes heterochromatin protein 1 beta (HP1β). These variants are situated within the chromodomain of HP1β, a critical region that mediates the interaction between HP1β and trimethylated H3K9. While a prior study identified three individuals with pathogenic *CBX1* variants, the variant and clinical spectrum of *CBX1* -related syndrome remains largely unknown. Here we report three patients with novel *de novo* heterozygous variants in *CBX1* . Patients with *CBX1* p.(Leu40Pro) and p.(Cys60Arg) manifested with global developmental delay, while the patient with *CBX1* p.(Cys60Gly) primarily exhibited speech delay without concurrent motor developmental delay. All variants reside within chromodomain of the HP1β protein. Interestingly, *in vitro* cellular assays revealed that *CBX1* p.(Cys60Gly) variant showed an intermediate cellular phenotype between the wild type and the *CBX1* p.(Leu40Pro) and p.(Cys60Arg) variants, suggesting a possible genotype-phenotype correlation. This report provides further evidence that missense variants within the chromodomain of HP1β cause a syndromic neurodevelopmental disorder, *CBX1* -related syndrome.

## Full Text Availability

The license terms selected by the author(s) for this preprint version do not permit archiving in PMC. The full text is available from the preprint server.

